# Nerve Fibers in Breast Cancer Tissues Indicate Aggressive Tumor Progression

**DOI:** 10.1097/MD.0000000000000172

**Published:** 2014-12-12

**Authors:** Di Huang, Shicheng Su, Xiuying Cui, Ximing Shen, Yunjie Zeng, Wei Wu, Jianing Chen, Fei Chen, Chonghua He, Jiang Liu, Wei Huang, Qiang Liu, Fengxi Su, Erwei Song, Nengtai Ouyang

**Affiliations:** From the Guangdong Provincial Key Laboratory of Malignant Tumor Epigenetics and Gene Regulation (DH, SS, XC, YZ, WW, JC, FC, CH, JL, WH, QL, FS, ES, NO), Medical Research Center, Sun Yat-Sen Memorial Hospital, Sun Yat-Sen University; Breast Tumor Center (DH, SS, WW, JC, FC, CH, JL, WH, QL, FS, ES); and Department of Pathology (XS, YZ, NO), Sun Yat-Sen Memorial Hospital, Sun Yat-Sen University, Guangzhou, China.

## Abstract

Emerging evidence has indicated nerve fibers as a marker in the progression of various types of cancers, such as pancreatic cancer and prostate cancer. However, whether nerve fibers are associated with breast cancer progression remains unclear.

In this study, we evaluated the presence of nerve fibers in 352 breast cancer specimens and 83 benign breast tissue specimens including 43 cases of cystic fibrosis and 40 cases of fibroadenoma from 2 independent breast tumor center using immunohistochemical staining for specific peripheral nerve fiber markers.

In all, nerve fibers were present in 130 out of 352 breast cancer tissue specimens, while none were detected in normal breast tissue specimens. Among 352 cases, we defined 239 cases from Sun Yat-Sen Memorial Hospital, Guangzhou, China, as the training set, and 113 cases from the First Affiliated Hospital of Shantou University, Guangdong, China, as the validation set. The thickness of tumor-involving nerve fibers is significantly correlated with poor differentiation, lymph node metastasis, high clinical staging, and triple negative subtype in breast cancer. More importantly, Cox multifactor analysis indicates that the thickness of tumor-involving nerve fibers is a previously unappreciated independent prognostic factors associated with shorter disease-free survival of breast cancer patients. Our findings are further validated by online Oncomine database.

In conclusion, our results show that nerve fiber involvement in breast cancer is associated with progression of the malignancy and warrant further studies in the future.

## Abstract 2

Supplemental Digital Content is available in the text

## INTRODUCTION

The tumor microenvironment comprises a variety of nonmalignant stromal cells that play a pivotal role in tumor progression and metastasis.^1–4^ Among these components, nerve fibers are emerging with great pathological value in many malignancies, including those of the pancreas,^5–7^ colon and rectum,^8^ prostate,^9^ head and neck,^10^ and biliary tract and stomach,^11^ although their role in tumor growth and progression remains unclear. Evidence from recent studies in pancreatic^12^ and prostate cancers^13^ has shown that nerve-derived molecules such as neurotransmitters and cytokines can enhance the malignant phenotype of cancer cells, including proliferation, cell survival, and invasiveness. On the contrary, cancer cells secrete neuromodulatory agents to induce neuroplasticity, neural invasion, and even neuropathic pain sensation.^14^ Therefore, a reciprocally interacting loop between nerves and cancer cells can be formed to promote cancer development. In organs abundantly innervated by nerve fibers, the tumor–nerve interaction seems to be an independent factor in the progression of pancreatic cancer and prostate cancer. However, whether nerve fibers also play an important role in breast cancer remains unclear.

In this study, we performed a detail immunohistological evaluation of the nerve fibers in specimens from 352 patients with breast cancer from different institutions. Our data showed that the thickness of nerve fibers was an important prognostic factor in breast cancer patients. Hence, nerve–cancer interaction may play an important role in breast cancer development, and blocking the interaction may lead to novel therapeutic approaches for breast cancer.

## MATERIALS AND METHODS

### Patients and Tissue Specimens

We used 352 formalin-fixed paraffin-embedded tissue samples from patients with primary ductal carcinomas of the breast in this study. For the training testing set, data were obtained from 239 female patients (median age 48.7 years, range 29–84) at Sun Yat-Sen Memorial Hospital from January 2003 to March 2010. Patients with breast cancer, and with clinicopathological characteristics and follow-up information available, were included. We included another 113 patients, with the same criteria as above, from the First Affiliated Hospital of Shantou University, Guangdong, China, between January 1, 2008, and May 30, 2012, in the independent validation set. Additionally, benign breast tissue samples were collected from 43 patients with cystic fibrosis of the breast and benign 40 patients with breast fibroadenoma. All of the samples were collected with informed consent according to the Internal Review and the Ethics Board of the Sun Yat-Sen Memorial Hospital of Sun Yat-Sen University.

### Immunohistochemistry

Paraffin-embedded samples were sectioned into 4-μm-thick slices. Antigen retrieval was performed using a pressure cooker for 30 minutes in 0.01 M citrate buffer (pH 6.0), followed by treatment with 3% hydrogen peroxide for 5 minutes. The specimens were incubated with antibodies specific for protein gene product 9.5 (PGP9.5), neurofilament (NF), and class III-β-tubulin overnight at 4°C. Immunostaining was performed using Diaminobenzidine according to the manufacturer's instructions. As a negative control, isotype-matched antibodies were applied.

### Specimens Analyzed

All specimens were serially sectioned transversely, and whole-mount histologic sections were examined by 2 of the authors. The presence of nerve fibers in breast cancer specimens was defined as carcinoma within the perineural space adjacent to a nerve. To quantify the presence of nerve fibers, the maximum diameter of the nerve fibers was measured with an ocular micrometer by using Nikon NIS-Elements BR software (Nikon, Melville, NY). We selected the optimum cutoff score for the diameter of nerve fibers in breast cancer using X-tile plots based on the association with the patients’ disease-free survival (DFS). X-tile plots provide a single and intuitive method to assess the association between variables and survival. The X-tile program can automatically select the optimum data cut point according to the highest χ^2^ value (minimum *P* value) defined by Kaplan–Meier survival analysis and log-rank test. We did the X-tile plots using the X-tile software version 3.6.1 (Yale University School of Medicine, New Haven, CT).

### Data Mining

The associations between PGP9.5 mRNA expression in tissue and the clinical features and outcomes of breast cancer were obtained using Oncomine Cancer Microarray database analysis (http://www.oncomine.org). Data were retrieved from the Oncomine web site. None of the studies at Oncomine showed contradictory results with statistical significance. Additional details of the study are available at Oncomine.

### Statistics

All statistical analyses were performed using Statistical Package for Social Sciences software for Windows Version 13.0 (SPSS, Chicago, IL). The χ^2^ test was applied to compare categorical data. Kaplan–Meier survival curves were plotted, and the log-rank test was applied. Groups of discrete variables were compared using the Mann–Whitney *U* test and the Kruskal–Wallis nonparametric analysis of variance. DFS was calculated as the time from the date of surgery to the date of the first recurrence or metastasis after surgery (in patients with recurrence or metastasis) or to the date of the last follow-up (in patients without recurrence and metastasis). Overall survival (OS) was calculated as the time from the date of diagnosis to the date of death or the date of the last follow-up (if death did not occur). The prognostic significance of clinical and pathologic characteristics was determined using univariate Cox regression analysis. Cox proportional hazards models were fitted for multivariate analysis. After the interactions between the variables were examined, a backward stepwise procedure was used to derive the best-fitting model. Both 1-sided and 2-sided tests were used for all statistical analyses and significance level was 0.05. We investigated the prognostic or predictive accuracy of the presence of nerve fibers using receiver operating characteristic (ROC) analysis. We used the area under the curve (AUC) at different follow-up times to measure prognostic or predictive accuracy.

## RESULTS

### Nerve Fibers Are Present in Breast Cancer

**FIGURE 1:**
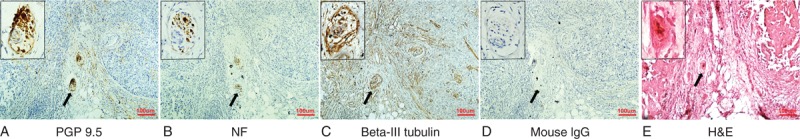
Nerve fibers present in breast cancer. (A) PGP9.5. (B) NF. (C) Class III-β-tubulin. (D) Isotype-matched antibody, mouse IgG. (E) H&E staining. Represented images of nerve fibers in breast cancer specimens. Nerve fibers were detected in serial sections of breast cancer tissues using IHC staining with 3 different specific neuronal markers. Original magnifications: 100× for the wild view; 400× for the left up corner. Scale bar, 100 μm. H&E = hematoxylin–eosin, IgG = immunoglobulin G, IHC = immunohistochemical, NF = neurofilament, PGP9.5 = protein gene product 9.5.

Most of previous studies examining the nerve fibers involvement in different types of cancer only used hematoxylin–eosin (H&E) staining.^15–17^ Although H&E staining can reveal the detailed structure of cancer specimens, immunohistochemical (IHC) staining with specific markers is more sensitive and specific than H&E staining to identify nerve fibers. To access the presence of nerve fibers in breast cancer, we examined 352 breast cancer specimens for the expression of specific neuronal markers, including PGP9.5, NF, and class III-β-tubulin, in serial sections. We found that these 3 markers demonstrated similar positive staining patterns in serial breast cancer sections, whereas the control isotype-matched antibodies demonstrated negative staining (Figure 1). Furthermore, nerve fibers identified by immunohistochemistry were validated by H&E staining in the serial sections showing a clearer histological structure of nerve fibers distributing in the tumor stroma (Figure 1). Therefore, nerve fibers are present in the stroma of breast cancer tissues.

### Nerve Fibers in Breast Cancer Tissues Correlate With High Malignancy

**FIGURE 2:**
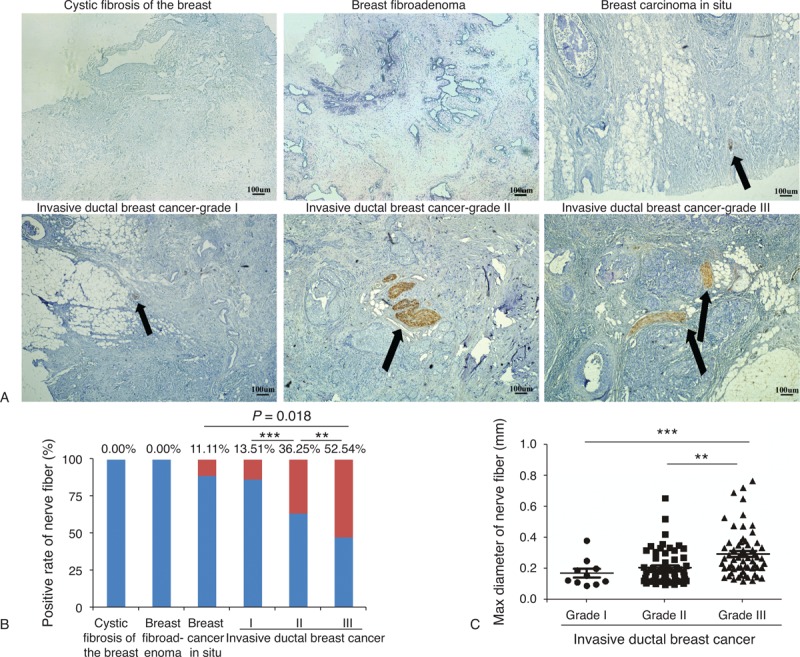
Nerve fibers in breast cancer correlate with high malignancy. Represented images of nerve fibers in different progression of breast tissue. (A) Nerve fibers were absent in cystic fibrosis of the breast and breast fibroadenoma, while they were present in breast carcinoma in situ and invasive ductal breast cancer. The arrow indicates the involvement of nerve fibers in breast tissue specimens. Scale bar, 100 μm. (B) The graph shows that the percentage of nerve fibers involvement in different progression of breast cancer tissues varied from 11.11% to 52.54%. Red bar represents the positive rate of the nerve fibers while blue bar represents the rate of absence of nerve fibers. *P* values were obtained using χ^2^ test. (^∗∗^: grade II vs grade III: *P* < 0.01 by both 1-sided and 2-sided test; ^∗∗∗^: grade I vs grade II/III: *P* < 0.001 by both 1-sided and 2-sided test; breast cancer in situ vs invasive ductal breast cancer: *P* = 0.018 by 1-sided test and *P* = 0.026 by 2-sided test.) (C) The maximum diameter of the nerve fibers in invasive ductal breast cancer samples also varied among cancer in situ and grades I–III cancers. (Mean + Standard error of mean; ^∗∗^: *P* < 0.01 by both 1-sided and 2-sided tests; ^∗∗∗^: *P* < 0.001 by both 1-sided and 2-sided tests.)

We next correlate the presence of nerve fibers, indicated by immunohistochemistry for 3 specific neuronal markers, with breast cancer progression in the patients. Among the 352 patients examined, nerve fibers were identified in 130 (36.93%) cases and were observed at the invasive front or in the center of the tumors, whereas no staining was observed in the adjacent nonneoplastic epithelia (Figure 2A and B). Additionally, nerve fibers were absent in all benign breast tissues, including fibrocystic lesions with or without atypical epithelial hyperplasia and benign breast fibroadenoma, whereas these fibers were occasionally identified in the stroma (2 out of 18 cases) of ductal carcinomas in situ (DCIS) of the breast (noncancerous tissue vs invasive breast cancer: *P* < 0.001 by both 1-sided and 2-sided tests; breast DCIS vs invasive breast cancer: *P* = 0.018 by 1-sided test and *P* = 0.026 by 2-sided test; Figure 2A and B). Furthermore, among the 130 cases with the presence of nerve fibers, the immunostaining for nerve fiber markers significantly differed among various histopathological gradings. The percentage of histopathological Grade III breast cancers with PGP9.5-positive immunostaining (52.54%) was higher than those of lower histopathological gradings (the positive rate of Grade II was 36.25% [58/160, *P* = 0.007 by 2-sided test, *P* = 0.005 by 1-sided test]; the positive rate of Grade I was 13.51% [10/74, *P* < 0.001 by both 1-sided and 2-sided tests, compared to Grade III and Grade II]; Figure 2B). Moreover, we further quantified the maximum diameter of nerve fibers in breast cancer tissues using Nikon NIS-Elements BR software. The mean diameter of the nerve fibers in Grade III breast cancer tissues was 331.2 μm, which was approximately 1.7 times greater than the mean diameter of the nerve fibers in Grade I tissues (176.8 μm, *P* < 0.001 by both 1-sided and 2-sided tests) and about 1.5 times greater than the one in Grade II tissues (212.6 μm, *P* < 0.01 by both 1-sided and 2-sided tests). However, there was no significant difference between Grade I and II breast cancers (*P* > 0.05 by both 1-sided and 2-sided tests; Figure 2C). Additionally, in the entire cohort, we found that the proportion of PGP9.5-positive cases was higher among high-graded primary tumors (*P* < 0.001 by both 1-sided and 2-sided tests), more lymph nodes metastasis (*P* = 0.007 by 2-sided test, *P* = 0.006 by 1-sided test,) and advanced clinical staging (*P* = 0.012 by 2-sided test, *P* = 0.009 by 1-sided test). However, there was no significant correlation between the presence of nerve fibers and the patients’ age, tumor size, and molecular subtyping (*P* > 0.05 by both 1-sided and 2-sided test) (see Table, Supplemental Digital Content 1, http://links.lww.com/MD/A78, which shows the correlation between the presence of nerve fibers in breast cancer specimens and clinical characteristics).

To quantitatively analyze nerve fibers in breast cancer, we used X-tile plots to generate the optimum cutoff value for the diameter of the nerve fibers in the training set (see Figure, Supplemental Digital Content 2, http://links.lww.com/MD/A78, which shows X-tile plots calculation). The figure shows the univariate analysis between diameter of nerve fibers and DFS (*P* < 0.001 by 2-sided test). Using X-tile plots, we included those patients with nerve fibers of diameter 0.21 mm or higher in the group at high risk of disease recurrence, and those with diameter <0.21 mm in the group at low risk of disease recurrence. The distribution of clinicopathological characteristics also varied between thinner (d ≤ 0.21 mm) and thicker (d > 0.21 mm) group. In the training set, thicker group was associated with higher histological grade (Grade III), greater tumor burden (T3–T4), more lymph nodes metastasis (N2–N3), higher clinical tumor node metastasis stage (Stages III–IV), and poorer prognosis (Table 1, left panel). In addition, we found that nearly half of the patients (42.9%) in the thicker group have triple negative breast cancer.

**TABLE 1:**
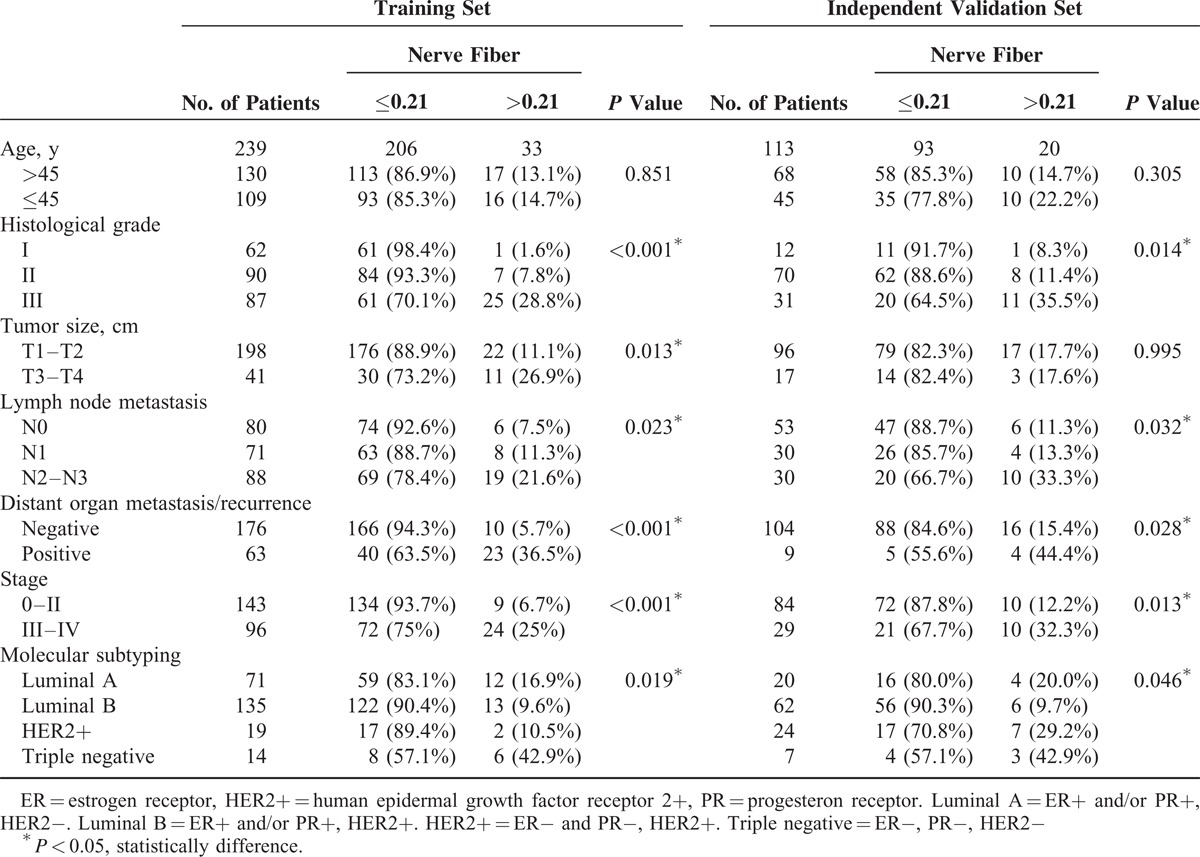
Correlation of the Thickness of Nerve Fibers With Clinicopathological Status in Training Cohort (239 Cases) and Validation Cohort (113 Cases) of Patients With Breast Cancer

To confirm that the diameter of the nerve fibers had similar prognostic value in different populations, we applied it to the independent validation set of 113 patients from different centers, classifying 93 (82.3%) patients as thinner group and 20 (17.7%) as thicker group. In the independent validation cohort, we obtained the similar results to the training set (Table 1, right panel).

To further validate these findings, we searched the Oncomine database for the expression of PGP9.5 in human breast cancer. Four datasets showed that the expression of PGP9.5 in breast cancer was higher compared to normal breast tissue, while 3 datasets showed an association between PGP9.5 expression and high-grade breast cancer with approximately 2-fold increase (Table 2 ). Furthermore, approximately 9 datasets showed that the expression of PGP9.5 was associated with metastasis, recurrence, or patient death during follow-up. The results found in the Oncomine database confirmed our findings that the involvement of nerve fibers is associated with breast cancer progression.

**TABLE 2:**
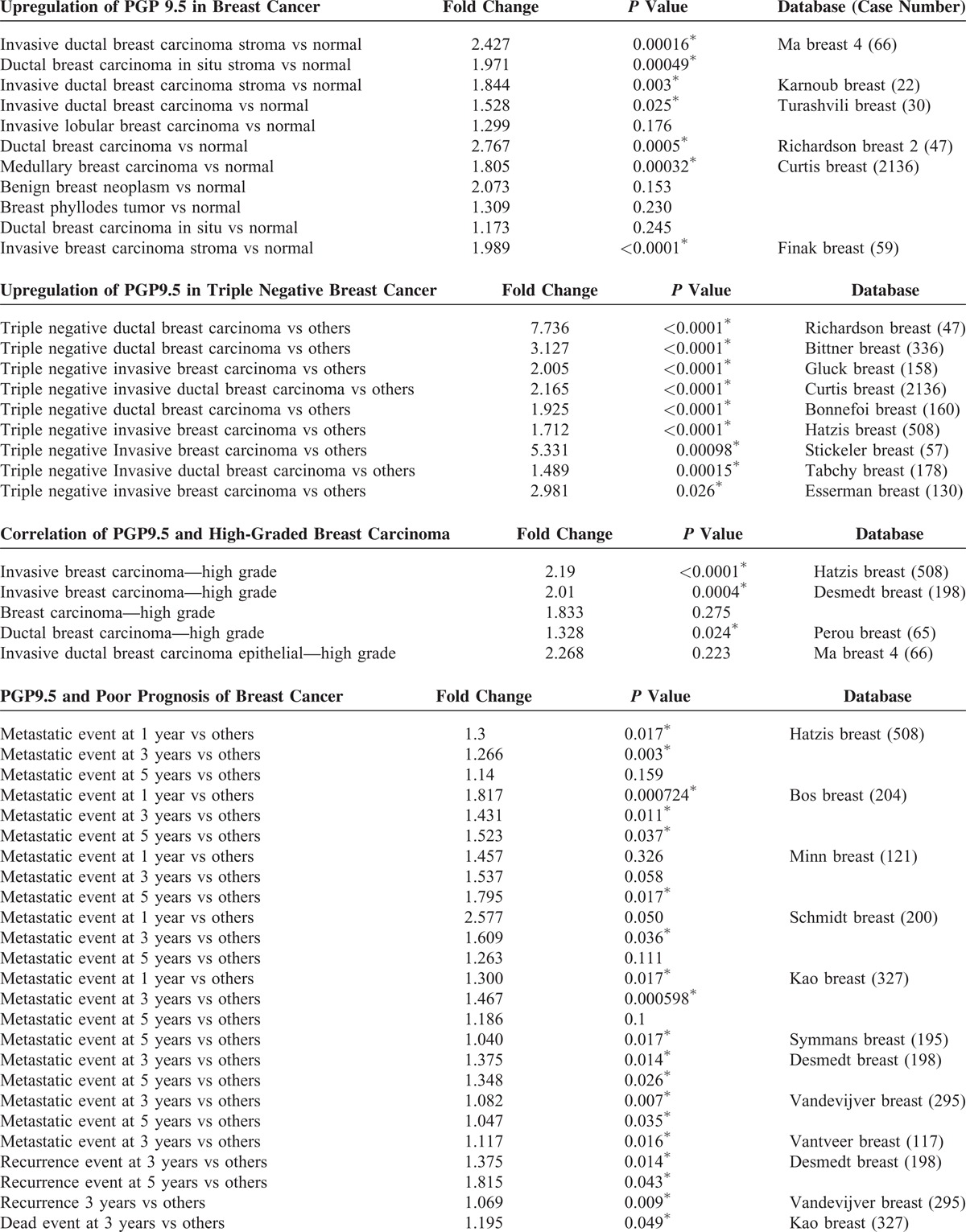
Clinical Features in Breast Cancer in Oncomine Online Database

**TABLE 2 (Continued):**
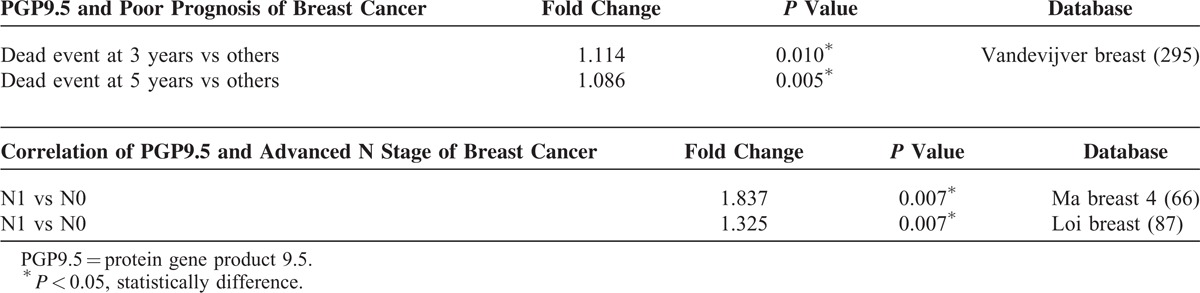
Clinical Features in Breast Cancer in Oncomine Online Database

### Thickness of Nerve Fibers Predict Prognosis for Breast Cancer Patients

**FIGURE 3:**
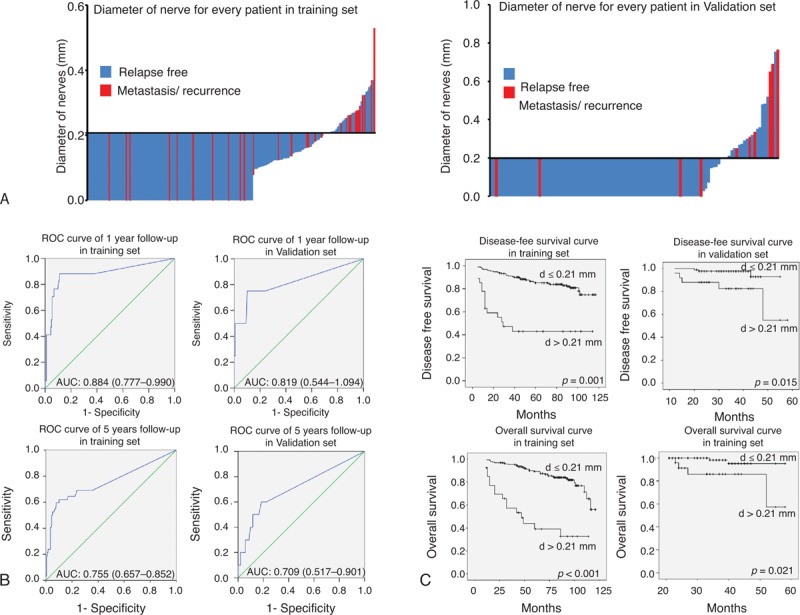
Diameter of nerve fibers can be a prognostic marker of breast cancer. The diameter of nerve fibers of every breast cancer patients in (A) training set (left) and validation set (right). (B) The cutoff value (d = 0.21 mm) was automatically generated by X-tile plots. Time-dependent ROC curves in the training set (left) and validation set (right). Data are AUC (95% CI) or hazard ratio (95% CI). Upper panel showed data of 1 year follow-up, whereas down panel showed data of 5 years follow-up. (C) Kaplan–Meier survival curve of DFS and OS in training set (left) and validation set (right). AUC = area under the curve, CI = confidence interval, DFS = disease-free survival, OS = overall survival, ROC = receiver operator characteristic.

Tumor recurrence and distant metastasis are responsible for poor survival of breast cancer patients. Therefore, we analyzed the prognostic value of PGP9.5 expression in the training cohort and the independent validation cohort using Kaplan–Meier analysis and log-rank test. In the training cohort, 40 out of 206 cases with thinner nerve fibers (d < 0.21 mm) developed local recurrence (15 cases) and/or distant recurrence (25 cases), whereas 23 of 33 cases with thicker nerve fibers (d > 0.21 mm) developed local recurrence (5 cases) and/or distant recurrence (18 cases) (Figure 3A, left panel). The median follow-up period for all patients was 84 months, ranging from 12 to 117 months. The breast cancer patients with thinner nerve fibers had a median DFS of 82 months, which was significantly longer than the 74-month DFS for patients with nerve fibers (*P* < 0.001 by both 1-sided and 2-sided tests). Breast cancer patients with thicker nerve fibers also demonstrated shorter OS (median: 82 months) compared to those with thinner nerve fibers (median 90 months; *P* < 0.001 by both 1-sided and 2-sided tests; Figure 3C, left panel).

We assessed the sensitivity and specificity of prognostic value of the diameter of nerve fibers with time-dependent ROC analysis at varying follow-up times (Figure 3B, left panel). The ROC curve analysis showed that diameter of the nerve fibers performed better in 1 year follow-up group (AUC = 0.884; 95% confidence interval [CI]: 0.777–0.990) than 5 years follow-up groups (AUC = 0.755; 95% CI: 0.657–0.852, Figure 3B). Similarly, these results were confirmed in validation cohort, as shown in Figure 3 A–C, right panel.

The results of univariate Cox regression analysis for DFS are shown in Table 3. In training cohort, DFS was significantly associated with tumor size, positive lymph node status, pathologic stage, histopathological grading, and the diameter of nerve fibers and human epidermal growth factor receptor 2 (HER2) status (*P* < 0.05 by 2-sided test). There was no significant association of DFS with age, estrogen receptor status, and progesterone receptor status (*P* > 0.05 by 2-sided test). In the multivariate analysis (Table 4), lymph node status, histological grade, the diameter of nerve fibers, and HER2 status were independent prognostic factors for DFS (*P* < 0.05 by 2-sided test). We also noted similar results in the independent validation set. Collectively, our data suggest that the thickness of the nerve fibers might serve as a previously unappreciated prognostic predictor of the long-term survival of breast cancer patients.

**TABLE 3:**
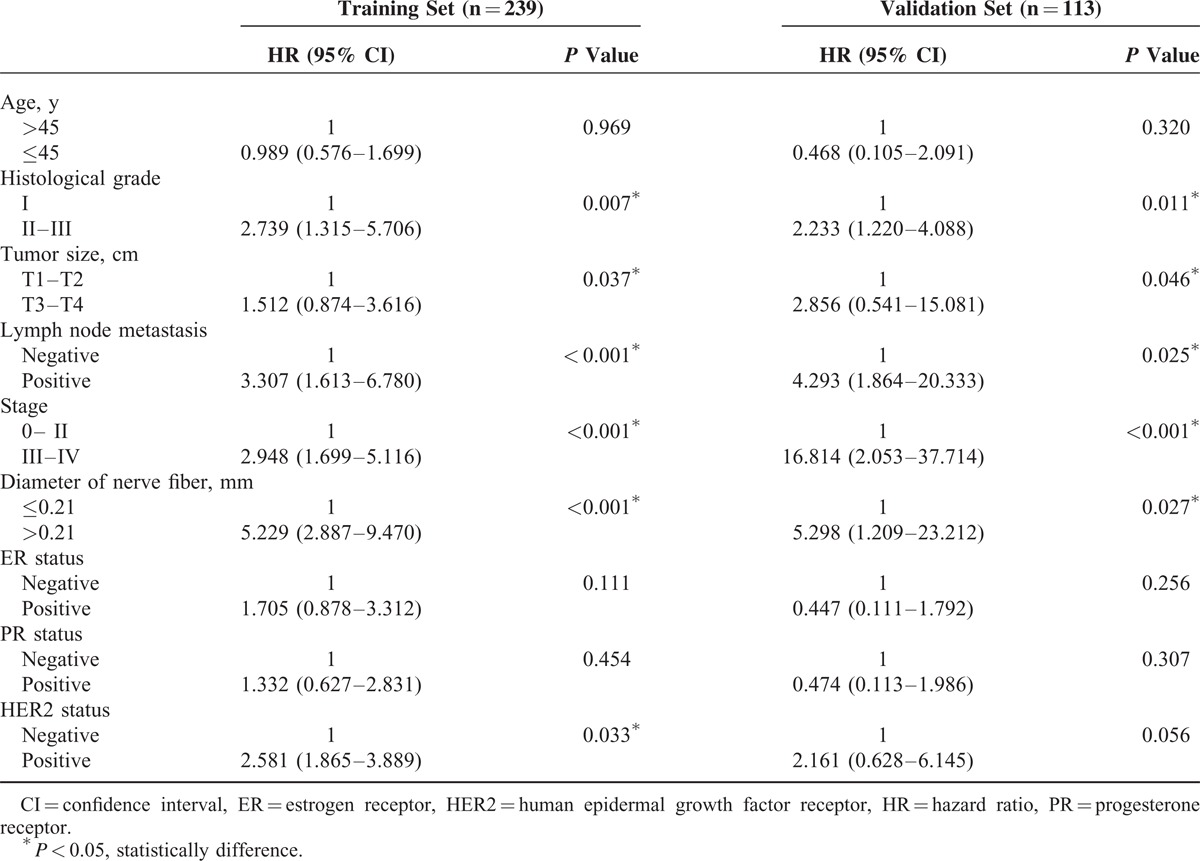
Univariate Cox Regression Analysis of Disease-Free Survival in Relation to Clinicopathologic Features

**TABLE 4:**
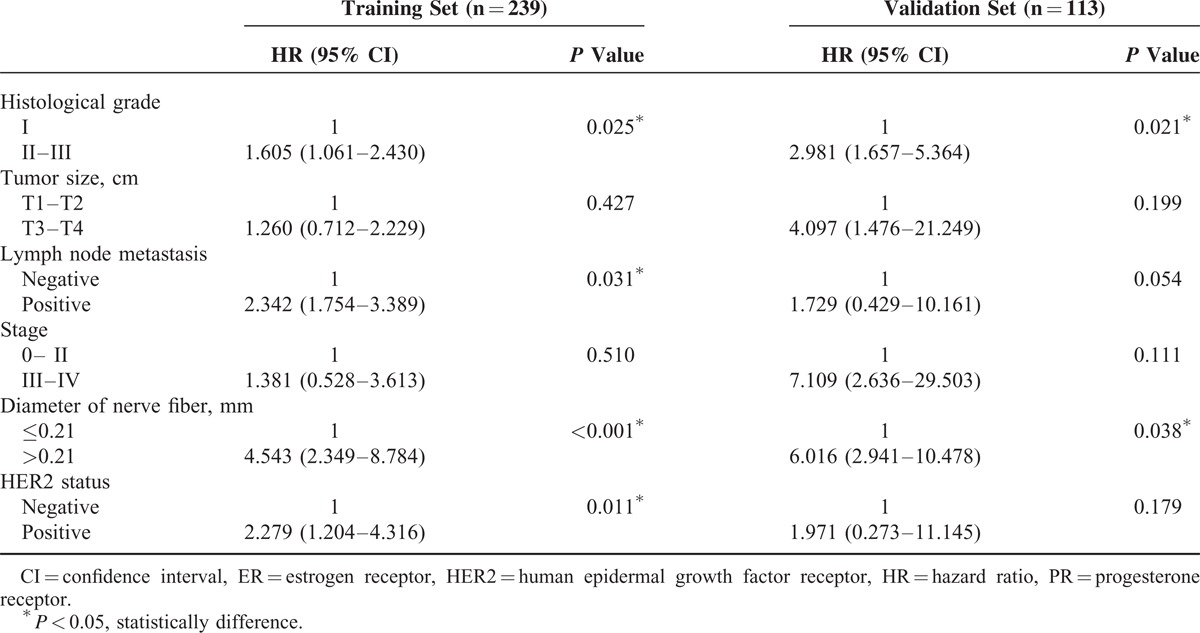
Multivariate Cox Regression Analysis of Disease-Free Survival in Relation to Clinicopathologic Features

### Nerve Fibers at the Invasive Front, But Not the Center of Breast Cancer Tissues Predict Poor Patient Outcome

**FIGURE 4:**
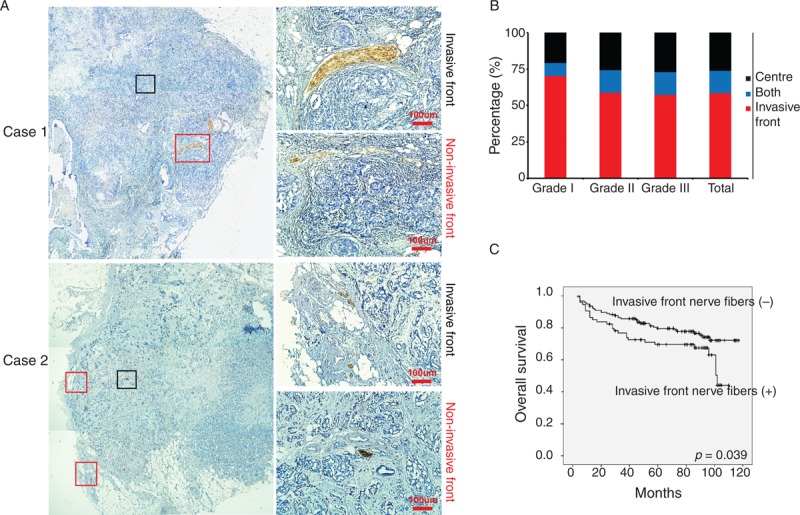
Nerve fibers in breast cancer specimens have different location. (A) Represented images of nerve fibers located in invasive front of breast cancer and the center of breast cancer. Original magnifications: left panel: 40×; right panel: 400×. Scale bar, 100 μm. (B) The proportion of nerve fibers located in invasive front and the center of tissue specimens from different grades of breast cancer. (C) Kaplan–Meier survival curve for patients with nerve fibers located in invasive front and the center of breast cancer.

We observed nerve fibers in 2 different locations within breast cancer specimens. In the entire cohort, nerve fibers were often observed at the invasive front (89/130) and less often observed in the center of the cancerous tissue (42/130; Figure 4A and B). The presence of nerve fibers in the invasive front was associated with high histological grading, positive lymph nodes metastasis, and distant organ metastasis/recurrence, while the presence of nerve fibers in the center of the cancer was only correlated with advanced histological grading (see Table, Supplemental Digital Content 3, http://links.lww.com/MD/A78, which illustrates the relationship between different location of nerve fibers in breast cancer specimens and clinical characteristics). Furthermore, patients with nerve fibers at the invasive front had shorter DFS as compared to patients without invasive front nerve fibers, whereas the DFS of patients with nerve fibers in the center of the cancerous tissue did not significantly differ from that of the other patient groups (Figure 4C). The different implications of nerve fibers between the 2 locations suggest that they may have unique functions during cancer progression.

## DISCUSSION

Nerve fiber involvement was reported to be associated with tumor progression in various malignancies (reviewed by Liebig et al^16^ and Marchesi et al^18^). Recent studies have shown that approximately 4% of breast cancer patients demonstrate nerve fiber involvement.^19^ However, the clinical significance and prognostic values of nerve fiber involvement in breast cancer remain unclear. IHC staining combined with H&E staining was more sensitive and specific to detect a specific cell type than H&E staining alone. Therefore, we assessed the presence of nerve fibers in breast cancer tissue by 3 specific peripheral neuronal markers including PGP9.5, NFs, and class III-β-tubulin. PGP9.5 is present in neurons, nerve fibers, and neuroendocrine cells in a variety of animal species.^20^ NFs are neuron-specific cytoskeletal components that allow nerve cells to establish and grow.^21^ Class III-β-tubulin is abundant in neuronal tissue,^22^ Kulchitsky neuroendocrine cells, and neuronal tumors,^23^ and associated with neuronal differentiation.^24^ But it was also reported in other cell types, such as breast cancer cells.^25^ Therefore, we identified nerve fibers by all the 3 markers (PGP9.5, NF, and class III-β-tubulin) stained positively. By using IHC and H&E staining, we found that nerve fibers were present in 130/352 cases (36.93%) of breast cancer. The higher rates of nerve fiber involvement in our studies compared to the previous studies suggest that IHC and H&E staining is more appropriate approach to detect nerve fiber involvement.

The neurotrophic factors secreted from cancer cells and other stromal cells promote the hypertrophy of nerve fiber in cancer, which reciprocally drive the cancer progression by producing various biological mediators. Therefore, we further evaluated the nerve fibers in breast cancer tissue by classifying it as thinner group (d ≤ 0.21 mm) and thicker group (d > 0.21 mm). The diameter of nerve fibers correlated with positive lymph node metastasis, high histological grade, and advanced clinical stage. More importantly, the thickness of nerve fibers in breast cancer is associated with worse DFS and OS independent of other conventional prognostic factors. Moreover, our findings were further validated by 19 online databases with information of breast cancer patients.

Previous studies^13^ have reported that tumor-infiltrating sympathetic fibers arising from normal prostate tissue play an important role in initial tumor growth, while intratumoral parasympathetic fibers can promote the proliferation and invasion of cancer cells. In our study, we also found that nerve fibers in breast cancer specimens were located at 2 distinct sites; they were often observed at the invasive front (89/130) and less frequently observed at the center of the cancerous tissue (42/130). The positive rate of nerve fibers at the invasive front was associated with high histological grade, positive lymph node metastasis, and poor prognosis, while the positive rate of nerve fibers at the center of the cancer was only correlated with advanced histological grade. The difference between these 2 types of nerve fibers suggests that they may have distinctive functions during cancer progression and warrant further studies in the future.

Randomized clinical studies have demonstrated that psychological distress in breast cancer patients can make tumors resistant to chemotherapy, and this process represents a significant reason for poor prognosis.^26,27^ Furthermore, these findings have been confirmed in cell culture studies and animal experiments.^28^ Although the mechanisms by which psychological distress affects the progression of breast cancers remain poorly understood, ample evidence has suggested that psychological stress can alter hormonal and neuronal secretions.^29^ These alterations can result in high levels of tissue catecholamine and adrenaline, which have a strong impact on the biological activities of breast cancer cells.^30^ In addition, chronic elevated levels of adrenaline and noradrenaline, which may increase tumor's invasiveness, have been reported in the plasma and urine of breast cancer patients.^31^ Moreover, recent retrospective clinical data suggest that patients with many malignant cancers, including prostate cancer,^32^ melanoma,^33^ and breast cancer,^34,35^ who take β-blockers, have a better prognosis and lower recurrence and mortality rates. In this study, we investigated this phenomenon in breast cancer patients and found that nerve fibers also exist in the breast cancer tissues and are associated with a poor prognosis in breast cancer patients. Therefore, our and other studies suggest that the nerve–tumor interaction may play an essential role in breast cancer progression and represent a potential therapeutic target for breast cancer.
